# Visualization Techniques in Healthcare Applications: A Narrative Review

**DOI:** 10.7759/cureus.31355

**Published:** 2022-11-11

**Authors:** Nehad A Abudiyab, Abdullah T Alanazi

**Affiliations:** 1 Health Informatics, King Saud Bin Abdulaziz University for Health Sciences, Riyadh, SAU; 2 Researcher, King Abdullah International Medical Research Center, Riyadh, SAU; 3 Researcher, King Saud Bin Abdulaziz University for Health Sciences, Riyadh, SAU; 4 Health Sciences, King Abdullah International Medical Research Center, Riyadh, SAU

**Keywords:** interactive visualization, visualization techniques, graphical presentation, big data, visualization

## Abstract

Nowadays, healthcare management systems are adopting various techniques that facilitate the achievement of the goals of evidence-based medical practice. This review explores different visualization techniques and their importance in healthcare contexts. We performed a thorough search on databases such as the SLD portal, PubMed, and Google Scholar to obtain relevant studies. We selected recent articles published between 2018 and 2021 on visualization techniques in healthcare. The field of healthcare generates massive volumes of data that require visualization techniques to make them easily comprehensible and to guide their efficient presentation. Visualization in healthcare involves the effective presentation of information through graphics, images, and videos. Big data systems handle a massive amount of information and require visualization techniques to present it in a comprehensible manner. The significance of visualization techniques in healthcare is not confined to healthcare practitioners and healthcare management but encompasses all the stakeholders; patients can benefit from the visualization of his/her data for a better understanding of their condition. In short, visualization techniques have demonstrated their benefits in the healthcare sector and can be extended to the payer and the patient. They have also had a positive impact on the quality of the healthcare provided as well as patient safety.

## Introduction and background

Data visualization in healthcare sectors relies on sophisticated modern technology that enables professionals from various fields to demonstrate their work and present information efficiently. Visualization techniques assist healthcare providers in understanding the trends that have occurred in the past as well as those in the present and in predicting and anticipating future trends and directions. Generally, data visualization involves representing data and information in various forms, such as graphs, charts, diagrams, and pictures [1]. These visualization techniques can provide healthcare providers with an easy way to identify and understand data trends, outliers, and patterns [2]. Visualization techniques have been essential in various healthcare sectors, especially in terms of supporting providers in making important clinical decisions regarding patient and community health. Through various visualization techniques, the healthcare organization can synthesize raw data into graphs and then present it in charts to enable the prompt interpretation of the trends and patterns [3].

This review aims to explore the different visualization techniques in healthcare, identify the benefits they bring to the field, and provide future directions for visualization-related studies.

## Review

Methods

This literature review provides a descriptive analysis of the application of visualization techniques in healthcare settings. The review criteria focus on incorporating the available studies in the review context and identifying recent research on visualization techniques in healthcare. The selected articles were obtained from various databases (SLD portal, PubMed, and Google Scholar).

Findings

The information about applications of visualization techniques in healthcare sectors is derived from data analysis and presentation. The most viable techniques are employed in the contemporary aspects of data visualization. Statistical analysis is complex to present via data visualization. Hence, it is accomplished through the use of interactive visualization. According to Gartner (2021), interactive visualization can be defined as manipulating graphical information via brightness, color, motion, and shape to elevate the meaning of the presented data [4]. Distinctly, the core objective of interactive and data visualization is to present and display the information in a way that the stakeholders will be able to interpret the data and increase their knowledge, thereby directly improving the service quality. In this context, many researchers have explored visualization techniques in healthcare sectors.

Historical Context of Data Visualization in Healthcare

One study discussed the application of data visualization in healthcare sectors in the context of what the author calls the Florence effect [5]. According to this study, the healthcare sector requires using the information in a potent way that propagates efficiency, promoting evidence-based practice [5]. The implication is that incorporating visualization techniques in healthcare sectors has enabled them to achieve various goals of evidence-based practice. Furthermore, the healthcare sector has established a platform that utilizes data visualization to interpret and assimilate complex healthcare data [5]. The researcher established the historical context of data visualization by referring to the efforts of Florence Nightingale in the 19th century [5]. Florence Nightingale was a military nurse who took care of injured British soldiers. Based on her daily treatment of injured soldiers, she accumulated sufficient patient information that could be used to help reduce the mortality of the other wounded soldiers. However, due to patient confidentiality and the strict rules regarding the disclosure of patient information, she designed a statistical representation through pie charts to present data that could help reduce mortality among military personnel. Later, it was discovered that statistical presentation made accessing and interpreting patient information and outcomes easier [5]. This study is crucial as it provides the historical context regarding the integration of data visualization in healthcare sectors and its importance in monitoring and evaluating healthcare indices.

Types of Data Visualization Techniques

According to the study by Narayan et al. in 2021, accumulating a large volume of healthcare data makes the big data concept very common in the healthcare sectors, considering the aspects of volume, velocity, variety, and veracity of the data [6]. Visualization techniques make big data less complex and easy to interpret, even for non-healthcare providers. The healthcare sectors need to have tools to develop visualization. Many tools use data visualization techniques, such as pivot tables and charts. Furthermore, different visualization tools are available for those with technical and non-technical backgrounds. For example, Microsoft Excel provides a great visualization tool for healthcare providers with little or no knowledge of technical aspects.

Another example is statistical software such as IBM SPSS and JASP. Statistical software provides interactive visualization systems that can be used by expert providers. These visualization tools can be used in public disease surveillance [6]. For efficient and prompt healthcare data visualization, it is essential to consider publicizing the information to the stakeholders via various platforms. In public community healthcare, sharing data and integrating visual information into one universal platform is beneficial and necessary. Several types of platforms are available for this purpose, including cloud-based platforms. Moreover, cloud-based platforms such as ParaView and Gephi enable the provider to generate and host the graphics. Furthermore, these platforms could facilitate scientific reproducibility by matching the scientific figures to their underlying data and promoting discussion among collaborators [6].

Process of Healthcare Data Visualization

Most of the time, data analysis in healthcare sectors incorporates control checks to maintain visualized data accuracy. Electronic health systems are rich in raw data that can be subject to analysis. Analytics data are fed into a learning machine, artificial intelligence tools, and other analysis tools [7]. These tools provide a visual presentation of healthcare data regarding different aspects, including disease prevalence, patient age that is associated with the most prevalence, and other factors related to the disease. After the analysis, the visual data can be contrasted with other data collected before, and the same analysis and visualization techniques can be applied to them. Also, this effort to contrast the data enables healthcare providers to easily identify the trends and the changes in different aspects that have occurred over a certain period [8].

Implementation of Data Visualization in Healthcare

The main objective of data visualization in healthcare sectors is to simplify complex data to make them user-friendly so that healthcare providers can easily interpret them.

Interactive Dashboard

**Figure 1:**
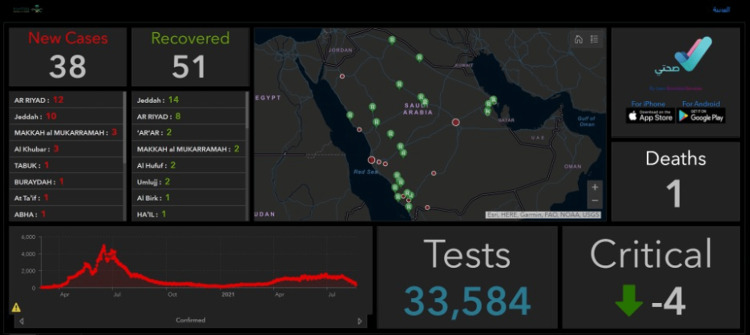
The dashboard used for analyzing COVID-19 prevalence in Saudi Arabia by the Ministry of Health COVID-19: coronavirus disease 2019

According to Pestana et al., dashboards and data analysis tools are usually built into the healthcare systems' existing software [9]. Usually, the dashboards help with combining several interactive reports. The dashboard is usually classified into three main types: the active type, which usually shows real-time data in healthcare organizations; the strategic type, which displays trends over time; and the analytical type, which presents advanced analytics. An example of data visualization through a dashboard in the Ministry of Health in Saudi Arabia is illustrated in Figure 1. The image shows the dashboard used for analyzing COVID-19 prevalence in Saudi Arabia

*Advantages of Visualization Techniques in Healthcare* Implementing and adopting different visualization techniques in healthcare sectors is vital in enhancing the overall healthcare provision by healthcare providers. Several healthcare facilities have implemented various data analysis tools in healthcare sectors, including machine learning and artificial intelligence. There are several benefits to implementing and adopting such techniques in healthcare facilities, and some of these are as follows: I. Improving overall patient care: utilizing health data visualization has positively affected the general provisioning of healthcare. Health data visualization has a significant role in supporting healthcare providers in their clinical decision-making and facilitating their ability to predict the threat and react immediately. Moreover, these threats were discovered by identifying various measures that permit the situation to be analyzed critically within a healthcare organization. Visualizing patient health data in real-time is crucial for improving the quality of care. It enables the healthcare provider to make the necessary clinical decisions based on the patient's situation [10]. For example, data visualization techniques help in monitoring various healthcare parameters, such as oxygen saturation [11]. Furthermore, patients' oxygen saturation levels and treatment can be analyzed in real-time to evaluate their responses to the treatment provided. For instance, during the coronavirus disease 2019 (COVID-19) pandemic, many patients developed lung disease due to severe pneumonia infection. Moreover, those patients were suffering from breathing difficulties and a decrease in their oxygen saturation level. Using data visualization techniques that involved displaying data on the monitoring machines was vital in assessing patients' responses to the oxygen level provided through the ventilator machine [12]. In various inpatient units, real-time data visualization has been used to monitor other patient parameters such as pulse, heart rate, and blood pressure [13]. Again, these real-time data visualizations significantly helped the providers to detect abnormal parameters and facilitate the intervention needed to improve patient outcomes and overall healthcare quality in the healthcare facility. II. Disease trend and pattern recognition: identifying trends and patterns is another significant advantage of utilizing visualization techniques in healthcare sectors. Determining the trends in healthcare is vital for making decisions regarding healthcare provision. Moreover, one of the critical trends in healthcare sectors is identifying and assessing disease patterns among specific populations. The trend of the disease patterns is an important attribute that should be monitored closely as it provides the necessary indications to investigate the factors causing an elevation in the trend. Data analysis and visual presentation on obesity is a good example of trend and pattern recognition; it acts as an indicator for public and community health. Identifying the factors behind the trends can help raise awareness about modifying the lifestyles of the patients as well as the general public [14]. III. Data presentation for various audiences: most of the time, healthcare data are more challenging to interpret among providers working in different disciplines compared to individuals with no medical background. Moreover, the primary purpose of data presentation is to simplify complex data so that it can be easily interpreted by any audience regardless of their background. An example of simplifying the data for disease prevalence is displaying it in graphics to the audience [1]. Also, utilizing the presentation to illustrate some of the factors affecting disease prevalence makes the medical data valuable and easily accessible to any intended audience. IV. Accelerated performance: Another advantage of real-time data visualization in a healthcare organization is accelerating the performance of the healthcare provided through several measures, such as ensuring prompt clinical decision-making in critical situations, which will positively impact patient prognosis and health status. Furthermore, accelerated performance can reduce the inadequacies of the provided care. Hence, accelerated performance can guarantee the overall better performance of healthcare organizations by building a good reputation for efficiency and better patient outcomes [2]. V. Errors and fraud detection: the most prominent benefit of implementing various data analytics and visualization techniques in healthcare sectors is that it enables the detection of frauds and errors that occur within healthcare organizations, such as errors and frauds in medical billing. According to the report published by Medicare and Medicaid, most of the fraud cases that occur within healthcare facilities are committed by the healthcare providers who work in the healthcare facility. Moreover, fraud cases usually cost healthcare facilities losses amounting to 58.5 to 89.3 billion dollars [14]. The most prevalent forms of billing fraud in healthcare facilities are duplicate billing, phantom billing, false prescription, and other types of insurance fraud by healthcare providers. Furthermore, clear and proper correlation among the stakeholders, including patients, healthcare payers, and providers regarding claims can enhance the integrity of the billing process and decrease fraudulent schemes [15]. Implementing data visualization techniques has significantly improved transparency in the healthcare sector.

Benefits of the review

Several advantages can be drawn from this literature review. It can be a source of information for researchers and students seeking knowledge on the same or similarly relevant topics. Moreover, the literature content has been designed in such a way that the audience can easily grasp and comprehend the information. In addition, the review can serve as a platform for identifying the loopholes in the application of visualization techniques in healthcare sectors based on recently published scientific research [16]. Also, it is essential to highlight that the review discussed several strategies that could help the medical professional improve the care delivered to the patient based on the best practices and applications of visualization techniques. It should be noted that some of the studies selected for this literature review are not directly about visualization techniques in the healthcare sector. We have made an effort to expand the scope of the review and explore the broader implications of the impact of visualization techniques.

Future directions

This review was based on recently published articles. It has certain drawbacks and loopholes that can be addressed by future research to enhance patient outcomes and quality management in healthcare sectors. There are some areas that need further investigation, including the use of interactive visualization techniques and their impact on healthcare sectors. In addition, only some of the studies included dealt with interactive visualization techniques, and studies have yet to analyze the importance of these techniques to healthcare facilities. Another area that needs further investigation is the role of patient-oriented visualization tools and their ability to support patients' health and outcomes [17]. Further studies need to be conducted by focusing on these topics, which would enhance and improve the knowledge base in terms of utilizing visualization techniques in healthcare sectors.

Limitations of the review

This review fully relied on published articles of the research that other scholars have conducted. Hence, our findings are wholly based on secondary knowledge obtained from peer-reviewed sources. These resources might include some errors that might impact the findings of this review as well.

## Conclusions

This review article highlighted the importance of data visualization techniques in healthcare from the standpoint of the following main benefits: improving the healthcare provided, prompt diagnosis of the disease, recognizing the patterns, simplifying the presentation of the healthcare data, accelerating healthcare performance, and improving error detection. The concept of visualization has been one of the significant innovations implemented and adopted in various healthcare facilities. Data visualization has several advantages and hence most healthcare facilities have embraced and implemented it in their day-to-day functioning. Moreover, the benefits of visualization techniques are clearly reflected in their effectiveness in the decision-making process, resulting in improved patient safety and quality of care. In addition, identifying the pattern and disease recognition via the presented data can provide vital knowledge in terms of treatment, diagnosis, and even adopting new policies in healthcare facilities. Also, it enhances the transparency of medical billing by reducing errors and fraud cases in healthcare facilities. Therefore, visualization techniques in healthcare sectors encompass many stakeholders, such as patients, healthcare practitioners, payers, and healthcare management. Also, we discussed the main limitations and challenges faced while conducting this literature review. Lastly, this review provides insight into the potential directions that future efforts on this topic can adopt.
